# Explaining rapid reinfections in multiple-wave influenza outbreaks: Tristan da Cunha 1971 epidemic as a case study

**DOI:** 10.1098/rspb.2011.0300

**Published:** 2011-04-27

**Authors:** Anton Camacho, Sébastien Ballesteros, Andrea L. Graham, Fabrice Carrat, Oliver Ratmann, Bernard Cazelles

**Affiliations:** 1 Laboratoire Eco-Evolution Mathématique, UMR 7625, CNRS-UPMC-ENS-AgroParisTech, 75230 Paris Cedex 05, France; 2 Universidade de Lisboa, Centro de Matemática e Aplicações Fundamentais, 1649-003 Lisboa, Portugal; 3 Department of Ecology and Evolutionary Biology, Princeton University, Princeton, NJ 08544-2016, USA; 4 UPMC—Paris 6, UMR-S 707, Paris 75012, France; 5 Inserm, UMR-S 707, Paris 75012, France; 6 Assistance Publique Hôpitaux de Paris, Hôpital Saint Antoine, Paris 75012, France; 7 Biology Department, Duke University, Durham, NC 27708, USA; 8 Department of Infectious Disease Epidemiology, Imperial College London, Norfolk Place, London W2 1PG, UK; 9 UMMISCO UMI 209 IRD—UPMC, 93142 Bondy, France

**Keywords:** influenza pandemic, reinfection, host immune response, state-space models, model selection

## Abstract

Influenza usually spreads through the human population in multiple-wave outbreaks. Successive reinfection of individuals over a short time interval has been explicitly reported during past pandemics. However, the causes of rapid reinfection and the role of reinfection in driving multiple-wave outbreaks remain poorly understood. To investigate these issues, we focus on a two-wave influenza A/H3N2 epidemic that occurred on the remote island of Tristan da Cunha in 1971. Over 59 days, 273 (96%) of 284 islanders experienced at least one attack and 92 (32%) experienced two attacks. We formulate six mathematical models invoking a variety of antigenic and immunological reinfection mechanisms. Using a maximum-likelihood analysis to confront model predictions with the reported incidence time series, we demonstrate that only two mechanisms can be retained: some hosts with either a delayed or deficient humoral immune response to the primary influenza infection were reinfected by the same strain, thus initiating the second epidemic wave. Both mechanisms are supported by previous empirical studies and may arise from a combination of genetic and ecological causes. We advocate that a better understanding and account of heterogeneity in the human immune response are essential to analysis of multiple-wave influenza outbreaks and pandemic planning.

## 1. Introduction

A swine-origin influenza A/H1N1 virus that arose in 2009 reminds us of the persistent risk of influenza pandemics. Lessons from the past are precious and may help us to anticipate and manage such potential disasters [1]. The most striking example is certainly the ‘Spanish’ influenza pandemic of 1918–1919 that occurred in three waves and caused about 50 million deaths worldwide in only nine months [2]. To date, this multiple-wave outbreak pattern, which has also been reported during several other pandemic episodes, remains only partially understood. On one hand, there is evidence from the 2009 A/H1N1 pandemic that climate variations and school closing and reopening shape the timing of successive epidemic waves [3]. On the other hand, the explicit reports of individuals experiencing reinfections over a short time interval during pandemic seasons are still poorly understood [4–7].

A commonly invoked hypothesis is that antigenically distinct, co-circulating influenza strains that confer only partial, humoral cross-immunity are each driving separate influenza outbreaks. Based on this assumption, Barry *et al.* [6] estimated the level of cross-protection between the first and the second waves of the 1918 H1N1 pandemic in US Army Camps and Britain, and Rios-Doria & Chowell [8] fitted a two-strain mathematical model to the 1918 H1N1 epidemic in Geneva. However, it is also commonly believed that evolving influenza strains may take years to escape population immunity, while the observed inter-wave periods are typically of the order of a few weeks [2]. Unfortunately, virus or serum samples from separate waves of past pandemics are too scarce to resolve this issue on empirical grounds.

Recent findings provide new evidence that supports the role of alternative reinfection mechanisms in driving multiple-wave influenza outbreaks. Notably, a large serological survey conducted during the first wave of the 2009 H1N1 pandemic highlighted host heterogeneity in the efficient development of humoral immunity [9]. This report challenges the assumption that influenza infection confers life-long protection against reinfection by the same strain [10]. From a theoretical perspective, it is possible to fit mathematical models in which individuals can be reinfected by the same strain to multiple-wave outbreaks [11,12]. However, these existing models are rather phenomenological and have been endowed with different biological interpretations, ranging from immune escape by antigenic drift to reinfection by immune deficiency [11,12].

**Figure 1.**
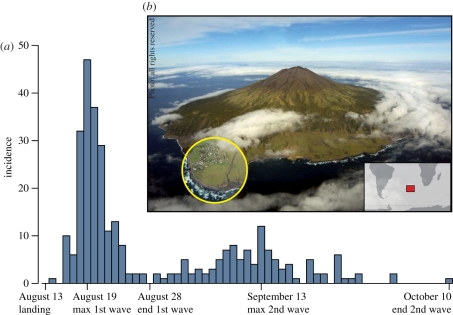
Historical and geographical data. (*a*) Daily incidence time series as it was originally reported by Mantle & Tyrrell [13]. (*b*) Photo and (inset) geographical position of the island of Tristan da Cunha in the South Atlantic Ocean. The Settlement of Edinburgh of the Seven Seas has been enlarged. Photographs from HMS Endurance's Helicopter taken on 12 April 2007.

What is lacking to direct further research is an evidence-based comparison of alternative immunological hypotheses that attempts to explain multiple-wave influenza outbreaks. We formulate six mechanistic stochastic models that incorporate a variety of potential antigenic and immunological mechanisms (i.e. positing both viral and host heterogeneity) that may explain rapidly occurring reinfection waves during influenza outbreaks. Particular emphasis is given to ensure that each hypothesis is associated with exactly one, parsimonious model. Using a simulation-based maximum-likelihood (ML) analysis, we interface these models with case data from the two-wave influenza epidemic that was reported on the remote island of Tristan da Cunha (TdC) in 1971 [13] (figure 1).

Historical circumstances make this case a unique natural experiment well-suited to mathematical modelling and statistical inference for multiple reasons: (i) moderate size and free social mixing of the community under study, (ii) full isolation of the community throughout the epidemic rules out the hypothesis that a second influenza virus was introduced from outside, (iii) incidents of influenza in the community were remarkably low and uniform before the 1971 epidemic, and (iv) the daily reported incidence counts are almost exhaustive [13] (§2*a*).

Our study reveals that, when demographic stochasticity is appropriately accounted for, the second epidemic wave can only be explained with mechanisms attributable to delayed or deficient humoral immune responses. Our strictly mechanistic interpretations enable us to quantitatively compare our results with empirical data and we close with an evaluation of the potential genetic and ecological determinants of variation in host susceptibility.

## 2. Material and methods

### (a) Data

TdC is a volcanic island in the South Atlantic Ocean. It has been inhabited since the nineteenth century and in 1971, the 284 islanders were living in the single village of the island: Edinburgh of the Seven Seas (figure 1*b*). Whereas the internal contacts were typical of close-knit village communities, contacts with the outside world were infrequent and mostly owing to fishing vessels that occasionally took passengers to or from the island. These ships were often the cause of introduction of new diseases into the population [14]. Focusing on influenza, several serological analyses between 1955 and 1963 provide important insights into the immune status of the adults among the 284 islanders present in 1971. Following an epidemic of A/H1N1 in 1954 during which most of the islanders were infected, antibodies to older influenza A and B types were detected in islanders over 20 years of age [15]. In 1961, when the volcano erupted, the island was evacuated to Britain and the islanders were given a polyvalent influenza vaccine that contained an A/H2N2 strain and a recent B strain. Serological studies showed a good response to this inoculation [16]. After the population returned to TdC in 1963, no influenza epidemic had been reported. In this context of a small population with a small and homogeneous immune repertoire against influenza virus, an unusual epidemic occurred in 1971, 3 years after the global emergence of the new subtype A/H3N2.

On August 13, a ship returning from Cape Town landed five islanders on TdC. Three of them developed acute respiratory disease during the 8 day voyage and the other two presented similar symptoms immediately after landing. Various family gatherings welcomed their disembarkation and in the ensuing days, an epidemic started to spread rapidly throughout the whole island population. After three weeks of propagation, while the epidemic was declining, some islanders developed second attacks and a second peak of cases was recorded. The epidemic faded out after this second wave and lasted a total of 59 days (figure 1*a*). Among the 284 islanders, 273 (96%) experienced at least one attack and 92 (32%, mainly adults) experienced two attacks. Only a few individuals experienced their single attack during the second epidemic wave. Unfortunately, only 312 of the 365 attacks (85%) are known to within a single day of accuracy and constitute the dataset [13]. A precise description of the clinical features of the illness as well as a review of the secondary infections were provided by Mantle & Tyrrell [13]. The authors reported that 85 per cent of the first attacks were moderate or severe, and this proportion decreased to 50 per cent for the second attacks. Serological analyses of 11 infected individuals demonstrated a high level of antibody against A/H3N2, a subtype to which the population had never previously been exposed. Moreover, seroconversion of individuals infected for the first time during either the first or the second epidemic wave attests that the virus was circulating throughout the epidemic. Unfortunately, no virological analysis was conducted to show whether first and second attacks were due to antigenically differing strains of A/H3N2.

### (b) Mechanistic modelling of reinfection hypotheses

In their original paper from 1973, Mantle & Tyrrell [13] proposed that this two-wave epidemic could have been caused by either the initial introduction of two separate viral agents or reinfection by the same viral agent. Although this second hypothesis appeared to them as the only possibility, they were unable to determine whether antigenic changes in the virus had occurred, allowing for second infection, or whether some patients did not acquire an efficient immune protection and either suffered a recrudescence of infection or were reinfected by other patients. We expand upon these possibilities as follows:

— Although originally dismissed [13], the first biological hypothesis (subsequently referred as the 2 Virus, or 2Vi, hypothesis) assumes that two separate viral agents, with different transmissibility, were introduced at the beginning of the epidemic.
— The Mutation (Mut) hypothesis assumes that a single initiating virus mutated within an infected host during the first epidemic wave, leading to the emergence of a new antigenic variant [8].
— The All-or-Nothing (AoN) hypothesis assumes that following recovery from infection, *some hosts* did not develop a long-term protective immunity and remained fully susceptible to reinfection by the same strain [9,11].
— The Partially Protective Immunity (PPI) hypothesis assumes that following recovery from infection, *all hosts* developed a long-term immunity that is not fully protective but reduces the risk of reinfection by the same strain [12].
— The In-Host (InH) hypothesis assumes that following infection some hosts were unable to completely eliminate the viral load and suffered from an intra-host recrudescence of infection [13].
— The Window-of-reinfection (Win) hypothesis assumes that following recovery, long-term protective immunity can take some time before becoming effective [9], resulting in a time window of susceptibility to reinfection by the same strain.

**Figure 2.**
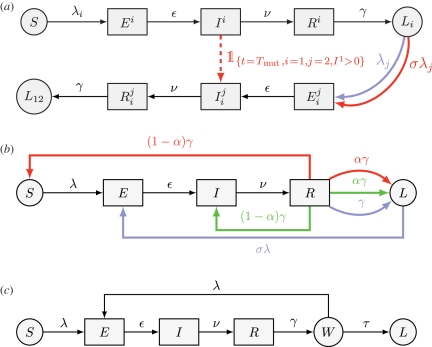
The six models with six different reinfection mechanisms can be retrieved by adding to three common skeletons (in black) the transitions corresponding to the indicated colour. All models have five epidemiological states in common: susceptible (*S*), exposed (*E*), clinically ill and infectious (*I*), temporarily removed from the transmission process (*R*) and protected in the long-term against reinfection by the same strain (*L*). To improve biological realism, durations of the states *E*, *I* and *R* are all gamma-distributed (electronic supplementary material, text S1.1). (*a*) The 2Vi (blue) and Mut (red) models implement a widely used history-based formalism [17,18] with (*i*,*j*) *ε* {1,2}^2^ and *i* ≠ *j*. Upper index stands for the infective strain, bottom index for the already-immunized strain, *λ*_*i*_ = *β*_*i*_(*I*^*i*^ + *I*_*j*_^*i*^)/*N* is the force of infection of strain *i* and both strains are supposed to have the same mean latent, infective and temporary removed periods (electronic supplementary material, text S1.2). Hosts recovered from strain *i* enter the *L*_*i*_ class and become completely protected against reinfection by strain *i* while remaining susceptible to the other circulating strain *j*. For the Mut model, the two strains are supposed to have the same transmissibility (*β*_1_ = *β*_2_, see electronic supplementary material, text S1.2) and to interact through a cross-immunity parameter *σ* *ε* [0,1] that acts by reducing the susceptibility to the other strain (electronic supplementary material, text S1.3). The dashed red arrow indicates that at time *T*_mut_ if *I*^1^> 0, one infectious host with the initial strain (*i* = 1) becomes infectious with the mutated strain (*j* = 2). (*b*,*c*) For the AoN, PPI, InH and Win models, *λ* = *βI*/*N* is the force of infection of the single strain. In the AoN model (red), we assume that hosts acquire full protection against reinfection with probability *α*, otherwise they re-enter the *S* class. In the PPI model (blue), we assume that all hosts develop long-term immunity that partially reduces the level of susceptibility through a protection factor *σ* *ε* [0,1]. In the InH model (green), we assume that infected hosts are able to clear the viral load with probability *α*, otherwise they suffer from an intra-host reinfection and, after some time, re-enter the *I* state. In the Win model (*c*), we assume host heterogeneity in the waiting time for acquisition of a completely protective immunity [9]: if *some* hosts re-enter the transmission process before protection is effective, they fall into a time window of susceptibility to reinfection (*W*). We simply assume that *all* hosts remain in the *W* state for a duration that is exponentially distributed: this distribution has a positive density in zero, thus enabling *some* hosts to immediately enter the *L* class (electronic supplementary material, text S1.4). Parameter descriptions can be found in figure 3. The transition rates to simulate the six stochastic models are provided in electronic supplementary material, text S1.5.

An extensively used epidemiological model for influenza dynamics is of susceptible–exposed–infectious–removed (SEIR) form [8,11]. After exposure to the virus, susceptible hosts (*S*) pass through an exposed state (*E*) of latent infection, become infective (*I*) and are finally removed (*R*) from the infectious pool as they simultaneously recover and acquire permanent immunity. However, our immunological hypotheses motivate a more accurate description of the different stages from infection to development of long-term protective immunity. We incorporate several known [8,11,12,17,18] and novel features to the classical SEIR model in order to mechanistically translate the six biological hypotheses into six stochastic state-space models (see figure 2 and electronic supplementary material, text S1, for further details). Particular emphasis is given to ensure that each model combines enough parsimony to enable parameter inference and enough complexity to match unambiguously to a single hypothesis.

### (c) Simulation and model selection

Given the small population of TdC, demographic stochasticity is expected to play a significant role in the epidemic dynamics, especially during the inter-wave period when the number of infected hosts is low and epidemic fade-out is likely to happen. We therefore used the stochastic framework of continuous-time Markov chains that naturally allows demographic stochasticity to be taken into account. The Markov chain events and the transition rates used to simulate the six models are provided in electronic supplementary material, text S1.5. Numerical simulations were performed using the exact algorithm provided by Gillespie [19]. Model-predicted incidence is computed by counting the daily number of new hosts entering the infectious class *I*. Since the dataset reports only 85 per cent of the total number of attacks and in order to take account of possible unreported asymptomatic cases, the observation process must also be modelled. More precisely, after having checked that the data were not overdispersed (electronic supplementary material, text S1.6), we assumed a Poisson process observation whose reporting rate parameter (*ρ*) was also inferred [20].

Our approach for evaluating the reinfection hypotheses rests on a statistical comparison of the corresponding state-space models to the shape and the dynamics of the observed daily incidence counts while, crucially, allowing for demographic stochasticity. For a time series *y*_1:*T*_ of *T* successive observations and a state-space model *H*_*i*_ with parameter vector *θ*, the likelihood is given by *ℒ*(*θ*|*H*_*i*_) = *P*(*y*_1:*T*_|*θ*, *H*_*i*_). Parameter inference and model selection are based on an iterated filtering procedure that converges to the ML parameter estimate (*θ*_ML_) for each model to the incidence data [20]. We performed log-likelihood profiles in order to check convergence to the ML and to calculate 95% confidence intervals for parameter estimates. Finally, we used the corrected Akaike information criterion (AIC_c_) to select the model that best explains the data: AIC_c_^*i*^ = −2*l*(*θ*_ML_|*H*_*i*_) + 2*k* + 2*k*(*k* + 1)/(*T* − *k* − 1), where *k* is the number of estimated parameters plus initial conditions, *T* = 59 is the number of observations and *l*(*θ*_ML_|*H*_*i*_) = log *ℒ*(*θ*_ML_|*H*_*i*_) is the maximized log likelihood. This correction accounts for the small sample size relative to the number of parameters (*T*/*k* < 10). Finally, we decomposed the maximized log likelihood of each model into conditional log likelihoods log *P*(*y*_*t*_|*y*_1:*t*−1_, *θ*_ML_, *H*_*i*_) in order to compare the six models at successive observation times *t* (see electronic supplementary material, text S2, for further details on the inference framework).

## 3. Results and discussion

### (a) Model selection

**Figure 3.**
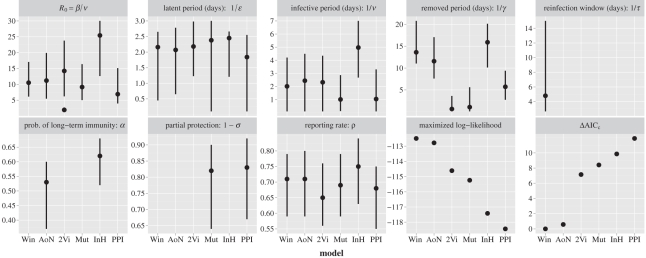
Maximum-likelihood estimates (dots) and 95% confidence intervals (vertical lines) for model parameters (see electronic supplementary material, table S2, for computational details). Numerical values are provided in electronic supplementary material, table S11, and we refer to electronic supplementary material, table S12, for initial conditions. The two different values of *R*_0_ for the 2Vi model correspond to the low and high transmissible viruses.

The maximized likelihood provides a first quantitative answer to the objective question: ‘How likely is it that the stochastic process resulted in the observed epidemic?’. The AIC_c_ [21] is then a related measure of the expected predictive capability of the model that penalizes model complexity. The rescaled AIC_c_ values, presented in figure 3, allow for an immediate ranking of the competing models and show that the Win mechanism best explains the data. Following the rough rule of thumb of Burnham & Anderson [22], the AoN hypothesis also receives substantial support (*Δ*AIC_c_ ≤ 2). In contrast, the 2Vi and Mut models have considerably less support (*Δ*AIC_c_ > 7), whereas the InH and PPI models have essentially no support (*Δ*AIC_c_ ≳ 10). This rule of thumb has proved to be efficient in many practical situations [22,23] and can formally be justified by computing the evidence ratio of each competing model (electronic supplementary material, text S3). However, it has also been shown that one should be cautious regarding the systematic use of this rule of thumb when applying AIC corrections [23], which motivates a more detailed analysis of *Δ*AIC_c_ values.

**Figure 4.**
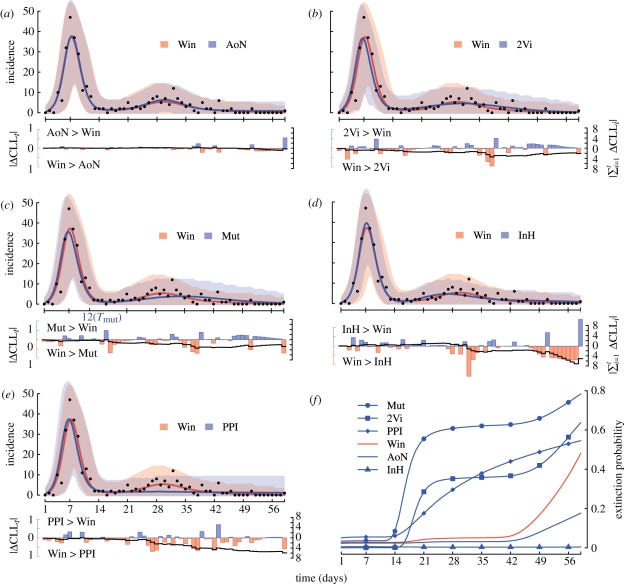
(*a*–*e*, upper panels) Qualitative comparison of the dynamics of each competing model (in blue: (*a*) AoN; (*b*) 2Vi; (*c*) Mut; (*d*) InH; (*e*) PPI) to the best model (Win, in red) and to the daily number of new cases reported in the data (black dots). For each model and associated ML parameter set, the mean predicted incidence (solid line) and corresponding 95% confidence envelope owing to demographic stochasticity and observation error (shaded area) were computed over 10^5^ stochastic simulations by conditioning on occurrence of the second epidemic wave (electronic supplementary material, text S4). Note that the dynamics of the Win and AoN models are superimposed (*a*). For the Mut model (*c*), emergence time of the second variant (*T*_mut_) is indicated on the *x*-axis. (*a*–*e*, lower panels) Quantitative comparison of the conditional log likelihoods for each observation time *t*: CLL_*t*_ = log(*P*(*y*_*t*_|*y*_1:*t*−1_, *θ*_ML_, *H*_*i*_ ), where *y*_1:*T*_ is the daily incidence dataset and *θ*_ML_ is the ML parameter set of a given model *H*_*i*_. Absolute differences (|*Δ*CCL_*t*_|, blue and red bars, left axis) allow quantitative identification of the parts of the time series where the Win model is better than the competing model *H*_*i*_ (Win > *H*_*i*_). Similarly, evolution of the absolute cumulative differences (|∑_*i*=1_^*t*^ *Δ*CCL_*i*_|, black line, right axis) indicates that the Win model performs always better than the competing model, at least after the second epidemic wave has begun. (*f*) Evolution of the extinction probability for each model defined at each point of time as the proportion of faded-out trajectories (*E*(*t*) = *I*(*t*) = 0) over 10^5^ stochastic realizations. (*f*) Blue line with circles, Mut; blue line with squares, 2Vi; blue line with diamonds, PPI; solid red line, Win; solid blue line, AoN; blue line with triangles, InH.

First, we identified that the differences in the log likelihoods (and thus AIC_c_) accumulate during the inter-wave period, the second epidemic wave and the extinction period after the second wave (figure 4*a–e*, lower panels). Second, to investigate whether the differences in AIC_c_ correspond to visible, practically significant differences in reproducing the characteristic second wave, we performed predictive checks by simulating 10^5^ time series under each ML-fitted model. Comparison of the behaviour of the best model (Win) with each competitor reveals superimposed dynamics with the AoN model and confirms that the dynamics of the four other models are different with respect to the second epidemic wave (figure 4*a–e*, upper panels). Furthermore, figure 4*f* shows that the extinction probability increases rapidly at the end of the first wave for the 2Vi, Mut and PPI models, whereas the Win and AoN models appear to be much more robust to stochastic extinctions during the inter-wave period. In the electronic supplementary material, text S4, we supplement a suite of statistical analyses to evaluate and compare the goodness of fit of these predictive simulations to the characteristic second-wave infection dynamics. Overall, these analyses support the view that, out of the models considered, the Win and AoN models explain the observed time series significantly better.

Our predictive simulations emphasize the paramount role of demographic stochasticity in the multiple-wave infection dynamics on this small island. In particular, given the elevated risk of epidemic fade-out during the low-prevalence inter-wave period, we find that only a stochastic framework can accurately assess alternative reinfection hypotheses.

Regarding the 2Vi hypothesis, *R*_0_ estimates (figure 3) indicate that the two viruses should have had very different transmissibility (both viruses are supposed to have the same duration of infection). The resulting dynamics reveal that during the first epidemic wave, the highly transmissible virus outcompetes the poorly transmissible virus, which has a 35 per cent risk of extinction owing to ecological interference (electronic supplementary material, text S5). However, if the poorly transmissible virus manages to maintain a low prevalence until the end of the first wave, when the highly transmissible virus goes extinct, then it can initiate the second epidemic wave (electronic supplementary material, figure S8).

On the other hand, the newly emerging variant in the Mut hypothesis has a 60 per cent chance of early extinction [24] because it has a low effective reproduction number *R*_e_ ≈ 1.7 (electronic supplementary material, text S5) and only one host initially infected. This latter choice can be justified *a posteriori*: the ML estimated level of antigenic escape is high (*σ*_ML_ ≈ 20% ) and similar to that of antigenic cluster transitions occurring each 2–8 years at the scale of the global human population [25,26]. However, we demonstrate in electronic supplementary material, text S6, that the AIC, the *σ*_ML_ and the risk of extinction are only weakly sensitive to the relaxation of this assumption.

The case of the PPI hypothesis is more complicated. A previous analysis of a similar but deterministic model [12] has revealed that dynamics depend on a reinfection parameter *σR*_0_. When this parameter is well above a reinfection threshold (*σR*_0_ > 1), reinfection becomes self-sustained and dynamics are SIS-like, whereas below this threshold primary infection dominates and leads to SIR-like dynamics. Our stochastic PPI model estimates *σR*_0_ = 1.18 and indicates critical dynamics: the reinfection parameter must be sufficiently high to reduce stochastic extinctions during the inter-wave period, but at the same time it must be sufficiently low to avoid sustained reinfection and therefore more than two epidemic waves. Put another way, epidemic fade-out after the second wave can only be reproduced near the reinfection threshold (*σR*_0_≳1), which simultaneously generates a significant inter-wave extinction probability.

The case of the InH hypothesis might seem surprising as its extinction probability remains null all along the epidemic. This result is in fact straightforward since reinfection in this model does not depend on a contact process and is not subject to demographic stochasticity. This reinfection mechanism is therefore very robust to the small population size but interestingly it is not supported by the statistical comparisons. This emphasizes the sensitivity and accuracy of our ML approach regarding the shape and the dynamics of the incidence time series (figure 4*d*).

Finally, it is remarkable that the two best models perform almost equally well despite being based on antagonistic mechanisms. Indeed, the Win hypothesis assumes that 100 per cent of the infected hosts can be reinfected during a limited period lasting an average of 4.8 days, whereas the AoN hypothesis assumes that only 47 per cent of the infected hosts can be reinfected at any time. This superimposed dynamics is in fact specific to the epidemiological context of TdC and we show in electronic supplementary material, text S7, that the dynamics of these two models would differ both qualitatively and quantitatively in the epidemiological context of a large population.

### (b) Parameter estimates

ML estimates and 95% confidence intervals for model parameters are shown in figure 3. The first observation concerns the high values of the basic reproduction number (*R*_0_) and its large variation from one model to another. *R*_0_ estimates are similar for the Mut, AoN and Win models (around 10), slightly lower for the PPI model, but more than twice as high for the InH model owing to an identifiability issue (electronic supplementary material, text S8). Overall, these high values for *R*_0_ are somewhat unusual: *R*_0_ is typically estimated around 2 for influenza, although exceptional cases have also been reported in closed populations [27]. We contend that a high value of *R*_0_ as well as rapid spread (the first peak was reached after only 6 days) and a high attack rate (96%) can be expected in small, isolated communities [28] without pre-existing immunity [29]. Furthermore, estimates of the effective reproductive number from the TdC incidence time series [30] are in agreement with our estimates of *R*_0_ (electronic supplementary material, text S9).

The generation time (average time between primary and secondary cases) can be estimated by the sum of the mean latent period plus half the mean duration of infectiousness [31]. ML estimates under the two best models are 3.2 days (Win) and 3.3 days (AoN) and in agreement with those previously published for A/H3N2 [32].

Recently, Mathews *et al.* [11] fitted a deterministic model (similar to the AoN model) on the same dataset. Their parameter estimates were very close to our values except for *R*_0_ (6.44 versus 11.27) and for the mean latent (1.36 versus 2.07 days) and infective (0.98 versus 2.44 days) periods. We suggest that this discrepancy is mainly attributable to the incorporation of demographic stochasticity in our approach. Indeed, deterministic models neglect the probability of stochastic extinction and should implicitly underestimate the above parameters that play a significant role during the inter-wave period (electronic supplementary material, figure S9).

The estimate of the proportion of the total cases that were reported in the data, *ρ*, is ≈ 70 per cent under all models. As expected, this value is under the empirical threshold of 85 per cent owing to data uncertainties (§2*a*) and the remaining discrepancy can easily be explained by undetected asymptomatic cases [11,32].

Variation of the mean temporary removed period from one model to another is expected since interpretation of the *R* state depends on the reinfection mechanism considered (electronic supplementary material, text S1.1). In particular, ML estimates under the two best models are 13.61 days (Win) and 11.57 days (AoN) and in agreement with the duration of the short-term, cell-mediated protection, as we now discuss.

### (c) Immunological support for reinfection

Our results suggest that heterogeneity among hosts (e.g. in the timely development of protective immunity) is a significantly more likely explanation for 1971's two-wave influenza outbreak on TdC than viral heterogeneity (e.g. in antigenic type). This suggestion finds empirical support in known mechanisms of immunity to influenza. In particular, both the Win and AoN mechanisms might be explained in light of genetic and/or ecological determinants of susceptibility.

A multi-pronged innate [33] and adaptive [34] immune response is optimal for clearing influenza infection. The innate response is the first to be activated and plays a key role through its ability to control early viral replication and to promote and regulate the virus-specific adaptive immune response [33]. Cytokines are among the most important bridges between the innate and adaptive responses to influenza [35]. The adaptive response itself may be broken into two critical sub-components: (i) the cellular immune response by which antigen-specific cytotoxic T lymphocytes (CTLs) eliminate infected cells and thus prevent viral release, and (ii) the humoral immune response by which serum antibodies efficiently neutralize the virus (both of which are promoted by T-cell help [34]). Antibodies can remain detectable for years after infection and prevent reinfection by the same strain as well as by sufficiently cross-reactive variants [36]. Genetic variation in any of these immune components might determine whether or how rapidly an individual develops protective immunity following primary influenza infection (in keeping with the AoN or Win hypotheses, respectively).

It is important to note that during primary influenza infection, the innate and cellular responses play the key role in viral clearance whereas neutralizing antibodies are generated later and do not play a significant role unless the viral load is high/sustained [37]. The primary CTL response is detectable in blood after 6–14 days whereas the neutralizing antibody response peaks at four to six weeks [38]. Critically, the CTL response is downregulated after viral clearance [37], disappears by day 21 post-infection [38] and is followed by a state of immunological ‘memory’ with antigen-specific T cells. The memory cells cannot prevent reinfection as well as specific antibodies could, but they can reduce the severity of the disease [37]. Together, these elements support the Win hypothesis: our parameter estimates indicate that the reinfection window occurred 17.8 days (s.d. 6.4 days) post-infection and lasted for 4.8 days (s.d. 4.8 days). This timing of susceptibility is in good agreement with the interval between the completion of CTL contraction and the full development of the neutralizing antibody response. Moreover, the reduced severity of most of the reinfection episodes in TdC (§2) might be explained by the T-cell ‘memory’.

In agreement with the AoN hypothesis, it has been reported that a protective serum antibody response cannot be detected in approximately 20 per cent of subjects after natural influenza infection [38]. However, our estimate is much higher and indicates that about 50 per cent of the islanders did not mount a protective response following the first infection. It has been proposed that this lack of protective immunity could be related to a low prior exposure to influenza [11]. Interestingly, the high level of consanguinity among the islanders, together with evidence that genetic bottlenecks occurred in the history of the population [39], may also have led to the over-representation of an unusual genotype involved in the control of influenza.

Furthermore, ecological factors including the dose of virus that initiates infection and the time interval between primary and secondary exposure may shape Win or AoN immunity, or indeed may make Win immunity appear as an AoN phenomenon. For example, the amount of virus in the lung determines the multiplicity of infection of innate antigen-presenting cells, which in turn affects their ability to induce subsequent adaptive responses [33]. Additionally, the rate at which immunity to reinfection develops is likely to interact with exposure rates to determine susceptibility. For example, when force of infection is high (as on TdC), many hosts are likely to be re-exposed to virus before their window of susceptibility to reinfection closes. If force of infection is low, then most hosts will have closed that window before re-exposure (electronic supplementary material, text S7).

### (d) Outlook

In this study, we assessed and compared six potential antigenic and immunological drivers of multiple-wave influenza A outbreaks on a two-wave influenza A/H3N2 epidemic that occurred on the island of TdC in 1971. We translated these hypotheses unambiguously into six mechanistic stochastic models, and employed a rigorous statistical framework based on ML [20] for parameter inference and model selection. In addition, we performed complementary statistical analyses, based on extensive simulations, to evaluate and compare the goodness of fit of the predictions of our six models. Our findings emphasize that a stochastic formulation is essential to capture demographic stochasticity induced by small populations [24] and/or low-prevalence inter-wave periods. We show that two mechanisms—both invoking host heterogeneity rather than viral heterogeneity—are significantly better supported by the data. Both mechanisms challenge the efficiency of the human immune response following primary influenza infection, indicating that, after a first attack, some individuals with delayed (Win) or deficient (AoN) humoral immune response could be reinfected by the same strain.

Further analyses to distinguish between the Win and AoN mechanisms will require more empirical data on reinfection at the individual level. Unfortunately, the original paper by Mantle & Tyrrell [13] does not provide such information, but surveillance of more recent influenza outbreaks may offer suitable data. For example, three cases of rapid reinfection by the same strain over a short time scale have been reported during the 2009 H1N1 pandemic [7]. We advocate application of state-of-the-art virological and immunological methods to samples from such cases. Alternatively, both parameter estimates and immunological support for the Win model indicate that successive infections by the same strain spaced over more than four to six weeks can only be explained by the AoN mechanism. We advocate application of our statistical approach to other multiple-wave datasets that occurred on a longer time scale than that of 1971's epidemic on TdC. It is also possible that ongoing outbreaks may enable tests of our results, and we refer to electronic supplementary material, text S7, for qualitative guidelines for such tests.

Finally, our results advocate a better account of host heterogeneity in the analysis of multiple-wave outbreaks. In particular, studies assuming that the immune response always provides a long-term humoral protection should overestimate the amount of immune escape required to sequential influenza variants to cause rapid reinfection [6] and multiple-wave outbreaks [8]. Put another way, our results may have important implications in the current context of influenza post-pandemic. Notably, the AoN mechanism, in addition to antigenic drift and compensatory mutations, would contribute to break population herd immunity by increasing the effective reproduction number of subsequent 2009 H1N1 influenza variants (electronic supplementary material, text S7). If empirically validated, these novel interactions should be included in epidemiological models aimed at pandemic planning and real-time risk prediction for influenza.
